# Ultraviolet optomechanical crystal cavities with ultrasmall modal mass and high optomechanical coupling rate

**DOI:** 10.1038/srep37134

**Published:** 2016-11-28

**Authors:** Wen Zhou, Zejie Yu, Jingwen Ma, Bingqing Zhu, Hon Ki Tsang, Xiankai Sun

**Affiliations:** 1 Department of Electronic Engineering, The Chinese University of Hong Kong, Shatin, New Territories, Hong Kong; 2 Shun Hing Institute of Advanced Engineering, The Chinese University of Hong Kong, Shatin, New Territories, Hong Kong

## Abstract

Optomechanical crystal (OMC) cavities which exploit the simultaneous photonic and phononic bandgaps in periodic nanostructures have been utilized to colocalize, couple, and transduce optical and mechanical resonances for nonlinear interactions and precision measurements. The development of near-infrared OMC cavities has difficulty in maintaining a high optomechanical coupling rate when scaling to smaller mechanical modal mass because of the reduction of the spatial overlap between the optical and mechanical modes. Here, we explore OMC nanobeam cavities in gallium nitride operating at the ultraviolet wavelengths to overcome this problem. With a novel optimization strategy, we have successfully designed an OMC cavity, with a size of 3.83 × 0.17 × 0.13 μm^3^ and the mechanical modal mass of 22.83 fg, which possesses an optical mode resonating at the wavelength of 393.03 nm and the fundamental mechanical mode vibrating at 14.97 GHz. The radiation-limited optical *Q* factor, mechanical *Q* factor, and optomechanical coupling rate are 2.26 × 10^7^, 1.30 × 10^4^, and 1.26 MHz, respectively. Our design and optimization approach can also serve as the general guidelines for future development of OMC cavities with improved device performance.

Optomechanical crystal (OMC) cavities that support high colocalization of optical and mechanical modes have opened a new era for coherent manipulation of photon–phonon interaction at the nanoscale^1^,^2^,^3^,^4^,^5^,^6^. OMC cavities based on a photonic crystal nanobeam structure^7^ support ultrahigh optical and mechanical *Q* factors, femtogram modal masses, gigahertz mechanical vibrations, and high optomechanical coupling rates^8^. They are highly desirable in the applications of resolved-sideband laser cooling^9^, optomechanically induced transparency^10^, high-speed acousto-optic devices^11^, and ultrasensitive signal detection^12^,^13^,^14^. Silicon has been the predominant platform for the implementation of OMC cavities^5^,^6^,^7^,^8^,^15^,^16^,^17^. In addition, wide-bandgap semiconductors can offer much more functionalities due to their broadband optical transparency, negligible nonlinear absorption, large Young’s moduli, and large piezoelectric effects. These materials (bandgap, Young’s modulus, refractive index) include aluminum nitride (AlN) (6.00 eV, 308 GPa, 2.20), diamond (5.50 eV, 1035 GPa, 2.40), silicon nitride (Si_3_N_4_) (5.00 eV, 310 GPa, 2.07), and gallium nitride (GaN) (3.40 eV, 330 GPa, 2.55). Among them diamond with the largest Young’s modulus is considered as a promising material for OMC cavities^18^, but its fabrication relies on an angled-etching technique which leads to asymmetric structural cross-sections and relatively low mechanical *Q* factors^19^,^20^. AlN, Si_3_N_4_, and GaN all have similar Young’s moduli, and OMC nanobeam cavities based on AlN^21^,^22^,^23^,^24^ and Si_3_N_4_^25^,^26^ have also been realized. In all the above implementations, it is difficult to obtain both a high optomechanical coupling rate and a small modal mass. Actually, the modal mass of all demonstrated OMC cavities with the optomechanical coupling rate greater than 1.00 MHz has been limited to above 85.00 fg^8^,^15^,^16^,^17^,^18^,^21^,^22^,^23^,^24^,^25^,^26^. This limitation is attributed to the reduction of the spatial overlap between the optical and mechanical modes. Since the volume of the optical mode is much larger than that of the mechanical mode, the system suffers from decreasing optomechanical interaction as the mechanical modal mass is reduced further.

In order to overcome the above limitation, we propose GaN-based OMC cavities operating in the ultraviolet regime. With the optical modal volume better matching the mechanical modal volume, such ultraviolet OMC cavities are capable of achieving an ultrasmall modal mass with a high optomechanical coupling rate. Compared with other wide-bandgap semiconductors, the prominent advantage of GaN is its bandedge emission covering the ultraviolet regime^27^ for integrating optomechanics and optoelectronics^28^ in a single device. Its mature epitaxial growth, composition, and doping technologies^29^ have led to high-power laser diodes^30^ and high-responsivity photodetectors^31^,^32^ based on InGaN/GaN multi-quantum-well structures. It has been employed for the realization of passive photonic integrated circuits^33^, ring resonators^34^, and two-dimensional photonic crystal cavities^35^,^36^. GaN could also be a promising platform for the development of active optomechanics^37^ in the ultraviolet regime, for studying the strong coupling between ultraviolet photons, GHz phonons, and electrons in the applications of high-speed, strong modulation of semiconductor lasers^37^ and enhanced mechanical ground-state cooling by optomechanical interaction^38^. In this paper, we design and numerically investigate GaN-based OMC nanobeam cavities, resonating at an ultrashort optical wavelength^8^,^18^,^26^,^39^ and vibrating at a high mechanical frequency^18^,^21^,^22^. We optimize the structure based on tuning the OMC mirrors for achieving ultrahigh optical and mechanical *Q* factors, with the optomechanical coupling rate greater than 1.00 MHz. In contrast to the previous implicit optimization methods^8^,^16^,^18^, our approach provides a step-by-step guideline and is advantageous to fine tuning of the overall device performance, which also applies to other wide-bandgap semiconductor platforms with similar refractive indices.

## Results

### Structural description and optimization procedure

**Figure 1:**
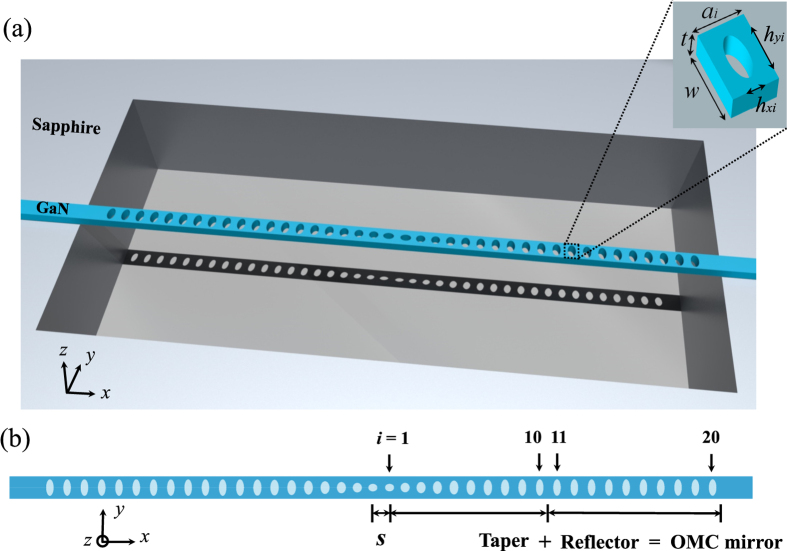
Schematic of an ultraviolet OMC nanobeam cavity in GaN. (**a**) Angled overview. The inset is a unit cell with dimension labels. (**b**) Top view. The OMC nanobeam cavity is constructed by joining two OMC mirrors with an adjustable cavity length *s*, while each OMC mirror consists of both a taper (*i* = 1–10) and a reflector (*i* = 11–20) region.

As shown in Fig. 1, an OMC nanobeam cavity supports both optical and mechanical modes in its center region because it provides an effective potential for the modes. Figure 1(a) shows an overview of the GaN-based OMC cavity. The top GaN nanobeam and the selectively etched sapphire substrate are marked in light blue and white, respectively. The elliptic air holes are introduced to engineer the potential for the optical and mechanical modes. These holes are arranged symmetrically along the nanobeam with respect to its center. Therefore, the OMC cavity can be modeled as a Fabry–Pérot cavity^40^, whose mirrors provide high reflection for light and sound simultaneously. Consequently, we may refer to one side of the hole array as an “OMC mirror”, whose reflection loss directly determines the intrinsic optical and mechanical *Q* factors. As shown in Fig. 1(b), an OMC cavity consists of two identical OMC mirrors, each of which consists of a taper and a reflector, with an adjustable cavity length at the center.

We aim at designing an OMC nanobeam cavity with the optical resonant wavelength around 400.00 nm, the mechanical modal mass around 20.00 fg, and the optomechanical coupling rate greater than 1.00 MHz. In principle, this is achievable through device downscaling and optimization in the entire parameter space, but we find it quite resource-demanding and time-consuming to find an OMC cavity with decent *Q* factors and optomechanical coupling rate simultaneously. Therefore, we have developed a new optimization procedure listed in the following four steps:

1. Designing the OMC mirror for obtaining high optical reflection in the targeted wavelength range and photonic and phononic bandgaps for the defect modes.
2. Obtaining high-*Q* optical and mechanical modes by varying the cavity length, during which the mismatch between the wavelengths of the optical mode and the OMC mirror’s reflection peak can be calibrated. By Steps 1 and 2 we should achieve an optical *Q* factor over 20 million and an optomechanical coupling rate around 0.80 MHz, which are important for the next fine-tuning steps.
3. Enhancing the optomechanical coupling rate to be greater than 1.00 MHz by decreasing all the lattice constants with the same factor, which improves the spatial overlap between the optical and mechanical modes.
4. Maximizing the optical *Q* factor by fine tuning the cavity length while maintaining the optomechanical coupling rate above 1.00 MHz. This step corrects the slight mismatch between the wavelengths of the optical mode and the OMC mirror’s refection peak introduced during Step 3 and obtains an overall optimized result of both the optical *Q* factor and the optomechanical coupling rate.

### Designing the OMC mirrors

The following parameters of GaN are used for designing the OMC naonbeam cavity. The wavelength (*λ*) dependence of the refractive index follows^41^


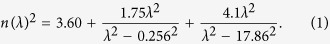


The density, Young’s modulus, and Poisson’s ratio are 6150 kg m^−3^, 330 GPa, and 0.183, respectively^42^,^43^. The thermal expansion coefficient, thermal conductivity, and heat capacity at room temperature (300 K) are 3.17 × 10^−6 ^K^−1^, 220 W m^−1 ^K^−1^, and 429.50 J kg^−1 ^K^−1^, respectively^44^,^45^,^46^.

We first design the OMC mirrors in order to obtain near-unity reflection for obtaining ultrahigh optical *Q* factors. We use elliptic air holes to construct the OMC mirrors for achieving high mechanical modal frequency. In the design illustrated in Fig. 1(b), we fix the total number of air holes to be 10 in both the taper and reflector regions, and use a quadratic taper for adiabatic mode transition with reduced scattering loss. The *i*-th (*i* = 1–20) unit cell has a lattice constant *a*_*i*_, with the *x*- and *y*-diameter of the elliptic air hole *h*_*xi*_ and *h*_*yi*_, respectively. The beam width *w* and thickness *t* are set to be 170 nm and 130 nm, respectively. The center-to-center separation between the two innermost air holes is denoted to be *s*, which can be used to tune the cavity length. In the taper region *h*_*xi*_, *h*_*yi*_, and *a*_*i*_ are quadratically varied from the 1st to the 10th unit cell. We preset both *h*_*y*1_ and *h*_*x*10_ to be 50 nm, an achievable feature size with typical electron-beam lithography and dry etching processes. We also set the lattice constant *a*_10_ to be 115 nm for obtaining a quasiphotonic bandgap at a wavelength around 400.00 nm. For *i* < 10, *a*_*i*_ is varied according to 115 × [1 − *C* × (9.5 − *i*)^2^] where *C* is 0.001565, the quadratic coefficient for achieving adiabatic mode transition. The parameters in the reflector region from the 10th to the 20th unit cell remain the same. Therefore, we will focus on *h*_*x*1_, *h*_*y*10_, and *s* for subsequent design and optimization.

**Figure 2:**
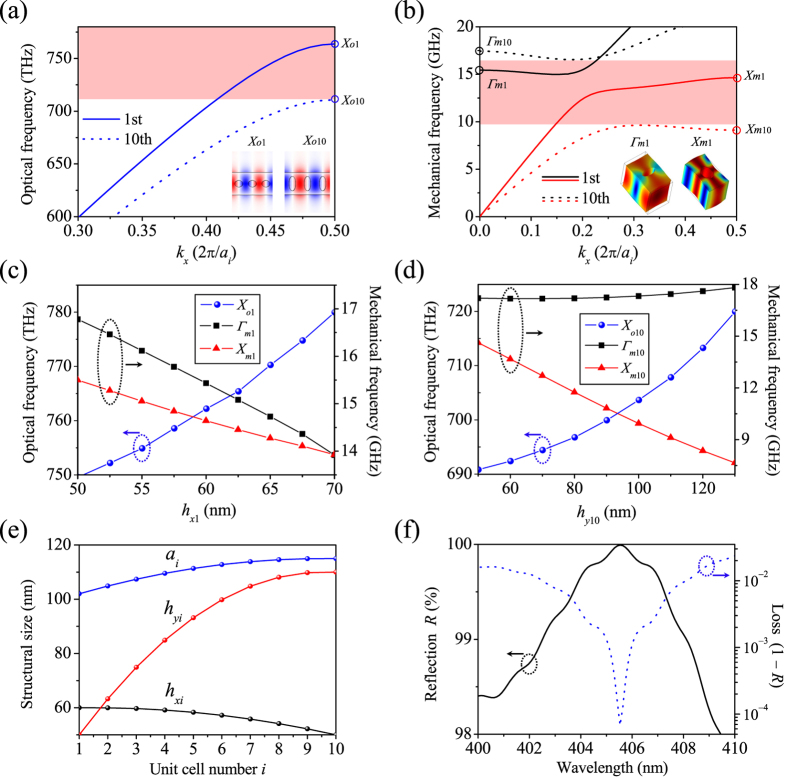
Designing high-performance OMC mirrors. (**a**) Photonic band diagrams of the 1st and the 10th unit cells. The pink-shaded region denotes the photonic bandgap of the 10th unit cell. Insets are the *E*_*y*_-field profiles at *X*_*o*1_ and *X*_*o*10_ for the 1st and the 10th unit cell, respectively. (**b**) Phononic band diagrams with *y*- and *z*-symmetry of the 1st and the 10th unit cells. The pink-shaded region denotes the phononic bandgap of the 10th unit cell. Insets are the displacement fields of the two mechanical modes at *Γ*_*m*1_ and *X*_*m*1_ for the 1st unit cell. (**c**,**d**) Optical modal frequency *f*_*o*_ at *X*_*o*1_ (*X*_*o*10_) and mechanical modal frequency *f*_*m*_ at *Γ*_*m*1_ (*Γ*_*m*10_) and *X*_*m*1_ (*X*_*m*10_) versus *h*_*x*1_ (*h*_*y*10_) for the 1st (10th) unit cell. (**e**) Assignment of *h*_*xi*_, *h*_*yi*_, and *a*_*i*_ (*i* = 1–10) for the unit cells in the taper region. (**f**) Spectra of reflection and the corresponding loss of the OMC mirror.

The photonic and phononic band diagrams of both the 1st and the 10th unit cell as shown in Fig. 2(a) and (b) are calculated using MPB^47^ and COMSOL Multiphysics^48^, respectively, to determine *h*_*x*1_ and *h*_*y*10_. The refractive index of GaN is set to be 2.55 in spite of a slight dispersion (*n* = 2.44–2.60) between 600.00 and 780.00 THz. In Fig. 2(a), only the dielectric bands are plotted because the air-band modes with photon energy higher than GaN’s electronic bandgap are highly lossy. The light cone is above and out of the zoomed band diagram region. The points with *k*_*x*_ = 0.50·(2π/*a*_*i*_), which are labelled as *X*_*oi*_ with *i* the numerical order of a unit cell, are used for determining the quasiphotonic bandgap and the localized photonic mode. The optical modal frequency (*f*_*o*_) at *X*_*o*1_ for the 1st unit cell is inside the quasiphotonic bandgap of the 10th unit cell, as indicated by the pink region. In Fig. 2(b), the phononic bands with *y*- and *z*-symmetry of the 1st and the 10th unit cell are provided. Here the points with both *k*_*x*_ = 0 and 0.50·(2π/*a*_*i*_), which are labelled as *Γ*_*mi*_ and *X*_*mi*_ respectively, are used for determining the quasiphononic bandgap and the localized mechanical mode. With *h*_*xi*_ decreasing and *h*_*yi*_ increasing, the mechanical modal frequencies (*f*_*m*_) at *Γ*_*m*1_ and *X*_*m*1_ shift respectively to the higher and lower side, thereby creating a large quasiphononic bandgap as indicated by the pink region. Figure 2(c) and (d) plot the respective optical and mechanical frequencies at *X*_*oi*_, *Γ*_*mi*_, and *X*_*mi*_ for the 1st and 10th unit cell as *h*_*x*1_ or *h*_*y*10_ varies. Since *f*_*o*_ of the air band at *k*_*x*_ = 0.50·(2π/*a*_*i*_) of the 10th unit cell is always larger than 799.92 THz with the varying *h*_*y*10_, *f*_*o*_ at *X*_*o*1_ of the 1st unit cell remains safely inside the quasiphotonic bandgap of the 10th unit cell. Additionally, Fig. 2(c) shows opposite trends for *f*_*o*_ and *f*_*m*_ with the varying *h*_*x*1_ of the 1st unit cell. In order to obtain both high optical and mechanical frequencies, we may choose *h*_*x*1_ to be 60 nm, with the corresponding *f*_*o*_ at *X*_*o*1_ 762.20 THz and *f*_*m*_ at *Γ*_*m*1_ 15.43 GHz. Lastly, in order to obtain both large quasiphotonic and quasiphononic bandgaps, we may choose *h*_*y*10_ to be 110 nm, with the corresponding *f*_*m*_ at *Γ*_*m*10_ and *X*_*m*10_ 17.44 GHz and 9.12 GHz, respectively. Therefore, the structural parameters (*h*_*xi*_, *h*_*yi*_, *a*_*i*_) for the 1st and the 10th unit cell have been determined to be (60, 50, 102) nm and (50, 110, 115) nm, respectively.

The structural parameters of the rest eight elliptic air holes in the taper region are determined by following a quadratic relation similar to that of *a*_*i*_. Here, the quadratic variation refers to the dependence of the structural parameters on the unit cell number *i*, which in principle allows for two types of parabolas—opening upward and downward. We have chosen the downward-opening type with the specific parameters shown in Fig. 2(e) for obtaining better performance, such as small optical modal volume, small mechanical modal mass, and high optomechanical coupling rate. With the structural design of the OMC mirror completed, we calculated its reflection and reflection-loss spectra as shown in Fig. 2(f). The reflection coefficient reaches the peak value of 99.99% at the wavelength of 405.53 nm, where the corresponding reflection loss is at least 30.7 times lower than that at 404.50 nm and 406.50 nm.

### Obtaining the high-*Q* optical and mechanical modes

We construct an initial design of OMC cavity by joining two identical OMC mirrors facing each other as shown in Fig. 1(b). We obtained the radiation-limited optical *Q* factor (*Q*_*o*_) by using MEEP^49^, where the material dispersion of GaN is incorporated. We also calculated the optical modal volume with the definition *V*_*o*_ = ∫ *ε*|**E**|^2 ^d*V*/max(*ε*|**E**|^2^). The simulated optical mode has a resonant wavelength of 405.66 nm with *Q*_*o*_ of 5.14 × 10^6^. The resonant wavelength is 0.13 nm away from that of the OMC mirror’s reflection peak, indicating room for further optimization of *Q*_*o*_. The round-trip loss can be reduced by a factor of 3.66 if the reflection loss is suppressed from 1.30 × 10^−4^ to the minimum 6.80 × 10^−5^.

The mechanical loss consists of two contributions: one is clamping (CL) loss due to leakage of the acoustic waves into the substrate, and the other is thermoelastic damping (TED) loss due to energy conversion and dissipation from the strain field into the temperature field^17^. Consequently, the total mechanical *Q* factor can be expressed as 1/*Q*_*m*_ = 1/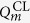 + 1/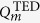. 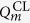 is usually proportional to the total number of air holes in the reflector. In our design 10 air holes are sufficient for obtaining negligible acoustic wave leakage.

The optomechanical coupling rate is defined as the cavity’s optical frequency shift induced by a mechanical zero-point displacement^5^. It can be calculated based on a perturbation theory^1^, by including contributions from the moving boundary (MB) and the photoelastic (PE) effects. The total optomechanical coupling rate is expressed as *g*_0_ = (*g*_MB_ + *g*_PE_)·(*ħ*/4π*m*_eff_*f*_*m*_)^1/2^, where *f*_*m*_ and *m*_eff_ are respectively the resonant frequency and modal mass of the mechanical mode. The moving boundary contribution *g*_MB_ is expressed as


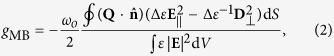


where *ω*_*o*_ is the optical resonant frequency, **Q** is the normalized displacement field, and 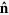 is the surface normal vector. *ε* is the material’s permittivity so that 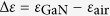 and 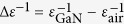. **E**_||_ and **D**_⊥_ are respectively the parallel component of the electric field and the perpendicular component of the electric displacement field with respect to the integral surface. The photoelastic contribution *g*_PE_ is expressed as


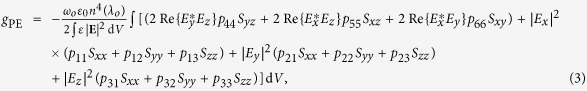


where *ε*_0_ is the vacuum’s permittivity, *n(λ*_*o*_) is the refractive index of GaN at the optical resonant wavelength, *S*_*ij*_ (*i*, *j* = *x*, *y*, *z*) is the strain field, and *p*_*mn*_ (*m*, *n* = 1–6) is the photoelastic coefficient of GaN in wurtzite structure^50^.

**Figure 3:**
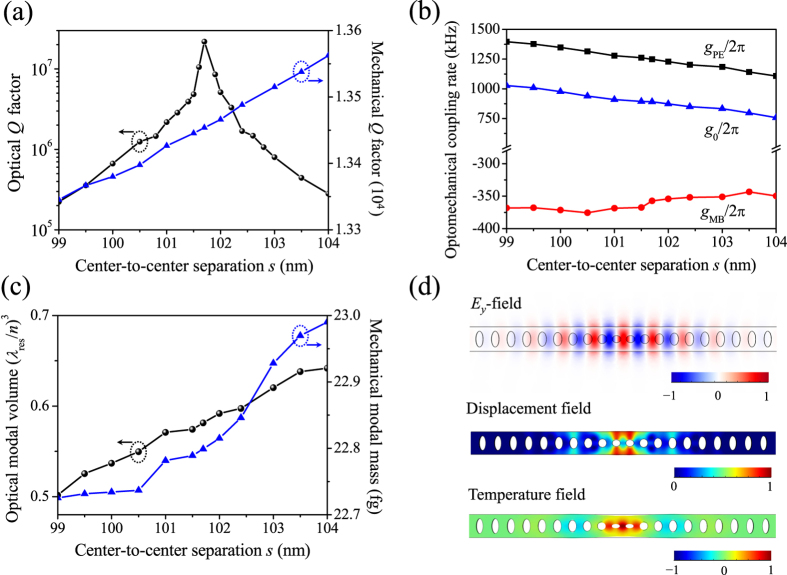
Designing high-*Q* optical and mechanical modes. (**a**–**c**) Effects of tuning the center-to-center separation *s* on the optical and mechanical *Q* factors (**a**), optomechanical coupling rates (**b**), and optical modal volume and mechanical modal mass (**c**). (**d**) Normalized *E*_*y*_-field, mechanical displacement field, and temperature profile at 300 K for the optimized OMC cavity with *s* = 101.70 nm.

For an as-constructed OMC cavity with the cavity length (center-to-center separation *s*) of 102.00 nm, we obtained *Q*_*o*_, 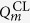, *Q*_*m*_, and *g*_0_/2π respectively to be 5.14 × 10^6^, 1.59 × 10^7^, 1.35 × 10^4^, and 873.95 kHz. We have an extra degree of freedom for optimization, i.e., tuning *s* around its initial value to get the maximal *Q*_*o*_. Figure 3(a–c) shows the results of the optical and mechanical *Q* factors, optomechanical coupling rates, and optical modal volume and mechanical modal mass as *s* varies from 99.00 to 104.00 nm. *Q*_*o*_ reaches the maximum when *s* is 101.70 nm, where the values of *Q*_*o*_, 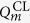, *Q*_*m*_, and *g*_0_/2π are 2.19 × 10^7^, 1.33 × 10^7^, 1.35 × 10^4^, and 890.88 kHz, respectively. These numbers meet the milestone prescribed in our design procedure and represent a good starting point for further optimization steps.

The optical resonant wavelength is 405.51 nm, which is now only 0.02 nm away from that of the OMC mirror’s reflection peak. The optical modal volume *V*_*o*_ is 2.34 × 10^−3 ^μm^3^ or 0.58·(*λ*_res_/*n*)^3^. The mechanical modal mass is 22.79 fg. The mechanical resonant frequency is 15.22 GHz and is well below the upper limit (16.54 GHz) of the quasiphononic bandgap, which results in extremely small clamping loss. Nonetheless, the total mechanical *Q* factor is largely limited by the TED loss under ambient temperature, yielding a *Q*_*m*_ three orders of magnitude lower than 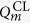 and the corresponding *f*_*m*_·*Q*_*m*_ product of 2.05 × 10^14^ Hz at 300 K. Figure 3(d) plots the normalized electric field, mechanical displacement field, and temperature profile of the optimized OMC cavity with *s* of 101.70 nm. It is easy to find that the optical mode and mechanical mode are colocalized in the center region with substantial spatial overlap.

### Enhancing the optomechanical coupling rate

In the last step, we obtained high *Q* factors, but the optomechanical coupling rate was below 1.00 MHz. A close examination of Fig. 3(b) reveals a monotonic relation between *g*_0_ and *s*: *g*_PE_ as the dominating term of *g*_0_ increases from 1108.14 to 1396.02 kHz when *s* reduces from 104.00 to 99.00 nm. This can be attributed to the increased overlap integral between the optical mode’s electric field *E*_*i*_ and the mechanical mode’s strain field *S*_*ij*_ as defined in Eq. (3).

**Figure 4:**
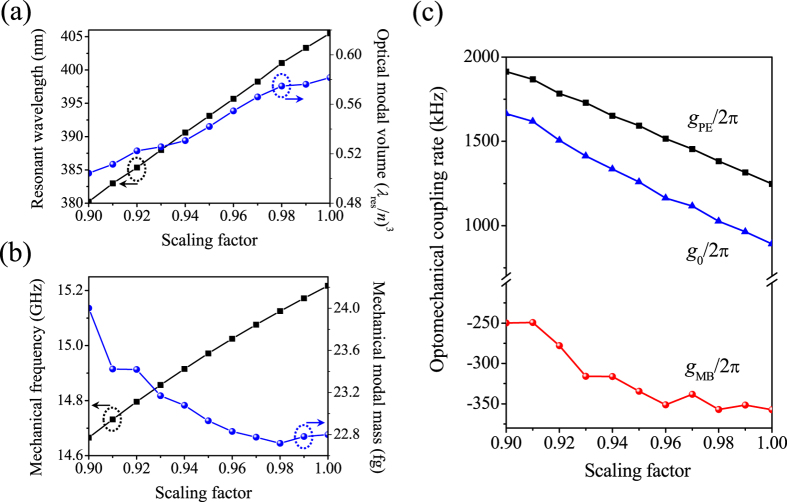
Enhancing the optomechanical coupling rate. (**a**–**c**) Effects of the downscaling of lattice constants on the optical resonant wavelength and modal volume (**a**), mechanical resonant frequency and modal mass (**b**), and optomechanical coupling rates (**c**).

In this optimization step, we enhance the optomechanical coupling rate by compressing all the lattice constants *a*_*i*_ by a scaling factor to increase the overlap between the electric field *E*_*i*_ and strain field *S*_*ij*_ (ref. 17). The effects of such an *a*_*i*_-downscaling process are straightforward in Fig. 4(a) and (b): when the scaling factor varies from 1.00 to 0.90, the optical modal volume reduces to 1.56 × 10^−3 ^μm^3^ or 0.50·(*λ*_res_/*n*)^3^, while the mechanical modal mass (volume) keeps increasing. The opposite dependence produces enhanced overlap between the optical and mechanical modes, yielding increased *g*_0_ as shown in Fig. 4(c). In the meantime, compression of all the lattice constants leads to larger values of *h*_*xi*_/*a*_*i*_, and consequently a shorter optical resonant wavelength as shown in Fig. 4(a). For example, the resonant wavelength shifts to 380.22 nm when the scaling factor is 0.90. Therefore, this downscaling process may induce a slight mismatch between the wavelengths of the optical mode and the OMC mirror’s refection peak although both are blue shifted. Figure 4(c) shows the results of the enhanced optomechanical coupling rates. For example, the total optomechanical coupling rate *g*_0_/2π reaches 1.26 MHz and 1.66 MHz when the scaling factor is 0.95 and 0.90, respectively. Therefore, one can choose a proper scaling factor for a desired *g*_0_/2π.

**Figure 5:**
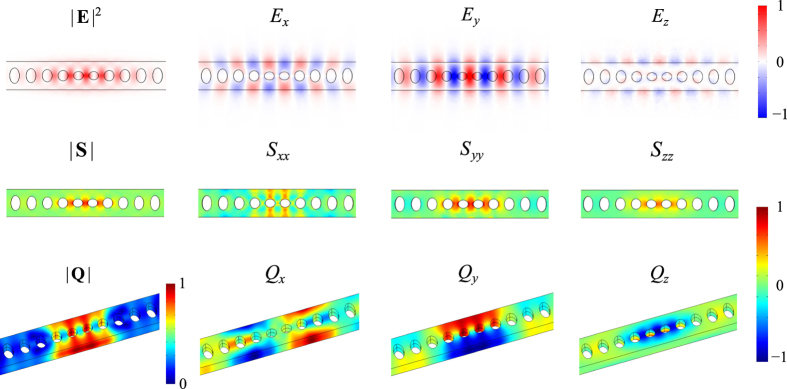
Profiles of the electric field E, strain field S, displacement field Q, and their individual components when the scaling factor is 0.90.

In order to better understand how the *a*_*i*_-downscaling process leads to the enhancement of *g*_0_, we list in Table 1 the individual contributions where *g*_PE*i*_ (*i* = 1–4) refers to the result calculated from the *i*-th term of the integrand in the right-hand side of Eq. (3). It is obvious that *g*_PE3_ which corresponds to the overlap between |*E*_*y*_|^2^ and (*S*_*xx*_, *S*_*yy*_, *S*_*zz*_) has the largest increment and is thus dominating. As a reference, we plot in Fig. 5 the spatial distributions of the electric field of the optical mode, and the strain and displacement field of the mechanical mode. Apparently, *E*_*x*_ and *E*_*z*_ are considerably weaker than *E*_*y*_, leading to very small overlap integrals for *g*_PE1_, *g*_PE2_, and *g*_PE4_. On the other hand, by comparing the profiles of |**E**|^2^ and |**S**|, one may identify that the optical modal volume is still slightly larger than the mechanical modal volume, indicating that the optomechanical coupling rate has further room for enhancement.

### Optimizing the *Q* factors while maintaining the high optomechanical coupling rate

**Figure 6:**
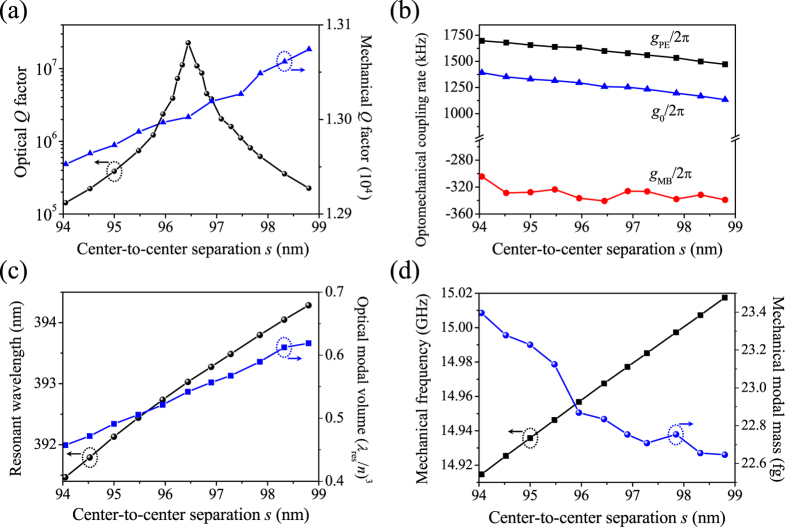
Maximizing the optical *Q* factor while maintaining a high optomechanical coupling rate. Effects of fine tuning the center-to-center separation *s* on the optical and mechanical *Q* factors (**a**), optomechanical coupling rates (**b**), optical resonant wavelength and modal volume (**c**), and mechanical resonant frequency and modal mass (**d**).

The last step of *a*_*i*_-downscaling induces different amounts of blue shift of the wavelengths of the optical mode and the OMC mirror’s refection peak and thus a slight mismatch between them, resulting in degradation of *Q*_*o*_. For example, when the scaling factor is 0.95 with the correspondingly updated *s* of 96.62 nm (0.95 × 101.70 nm), the resonant wavelength blueshifts to 393.13 nm and *Q*_*o*_ drops from the previously optimized 2.19 × 10^7^ to 1.09 × 10^7^. Meanwhile, the reflection peak of the compressed OMC mirror blueshifts to 392.98 nm. Similar to the implementation in Step 2, we may retrieve the high *Q*_*o*_ by fine tuning the cavity length. The purpose of this fine-tuning step is to enhance *Q*_*o*_ substantially at little expense of *g*_0_ reduction. Figure 6 shows the variation of all the previous simulated properties with the cavity length *s* tuned around 96.62 nm. When *s* is readjusted to 96.44 nm (0.95 × 101.52 nm), *Q*_*o*_ reaches the maximum again, with *Q*_*o*_, 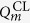, *Q*_*m*_, and *g*_0_/2π being respectively 2.26 × 10^7^, 1.18 × 10^7^, 1.30 × 10^4^, and 1259.12 kHz. The corresponding optical resonant wavelength, optical modal volume, mechanical resonant frequency, and mechanical modal mass are respectively 393.03 nm, 0.54·(*λ*_res_/*n*)^3^, 14.97 GHz, and 22.83 fg. A comparison of these parameters with those in Step 2 concludes that the optical modal volume is reduced from 0.58·(*λ*_res_/*n*)^3^ to 0.54·(*λ*_res_/*n*)^3^ while the mechanical modal mass is increased from 22.79 to 22.83 fg, which contributes to a better modal overlap and a high optomechanical coupling rate above 1.00 MHz.

It should be noted that *g*_0_/2π maintains greater than 1.00 MHz in the entire *s* tuning range in Fig. 6(b), indicating the existence of a sufficiently wide window for fine tuning the optical resonance. We also simulated OMC cavities of even smaller scaling factors. When the scaling factor is 0.92, the optimized OMC cavity resonating at optical wavelength 385.19 nm, mechanical frequency 14.79 GHz possesses *Q*_*o*_, *Q*_*m*_, *m*_eff_, and *g*_0_/2π of 1.29 × 10^7^, 1.28 × 10^4^, 23.36 fg, and 1539.97 kHz, respectively. When the scaling factor is 0.90, the optimized *Q*_*o*_ can still reach a high value 1.02 × 10^7^, with the corresponding resonant wavelength as short as 380.16 nm and *g*_0_/2π as high as 1805.88 kHz.

## Discussion

Table 2 compares the simulated performance of our GaN-based OMC nanobeam cavities with those of the state-of-the-art designs^8^,^18^. The design in this work has a shorter optical resonant wavelength 393.03 nm, a higher mechanical frequency 14.97 GHz, and a smaller modal mass 22.83 fg. The modal mass is comparable with the designed values of the world’s smallest optomechanical systems based on the concept of NEMS-in-cavity^51^,^52^. The large optomechanical coupling rate 1.26 MHz facilitates obtaining a large cooperativity for strong photon–phonon interaction^5^. In addition, the design and optimization approach we have developed is intuitive and straightforward, by avoiding searching from a large parameter space of numerous realizations.

**Figure 7:**
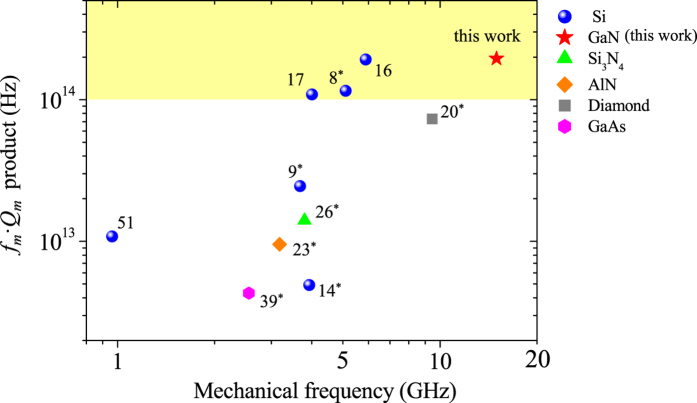
Plot of *f*_*m*_·*Q*_*m*_ product (300 K) with mechanical frequency of OMC nanobeam cavities in various materials. The *f*_*m*_·*Q*_*m*_ value of 1.95 × 10^14^ Hz from this work is in the 10^14^ Hz region (yellow shaded) along with three other works (refs 8,16,17). References with a star in the superscript represent experimental results.

A good figure of merit for evaluating different OMC cavities is the *f*_*m*_·*Q*_*m*_ product^5^,^53^,^54^,^55^,^56^ due to the trade-off between *f*_*m*_ and *Q*_*m*_^57^. Since *Q*_*m*_ is temperature dependent, it is important to compare all of them at the same temperature, i.e., 300 K. Therefore, we estimated the *Q*_*m*_ at 300 K based on their values in refs 8,9,16 and the relation 
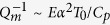
, where *E*, *α*, *T*_0_, and *C*_*p*_ are the Young’s modulus, the thermal expansion coefficient, the equilibrium temperature, and the heat capacity, respectively^58^. From the comparison in Fig. 7, one may notice that this work with a *f*_*m*_·*Q*_*m*_ product of 1.95 × 10^14^ Hz represents a high value along with three other works (refs 8,16,17) in the 10^14^ Hz regime. It should also be noted that the mechanical frequencies of previous OMC nanobeam cavities are all below ~10 GHz (ref. 18 not included due to the unavailability of *Q*_*m*_), because of the relatively large device size for supporting the near-infrared optical resonances (*λ*_res_ ≥ 980 nm). It is clear that an ultraviolet OMC nanobeam cavity investigated in this work provides a way for obtaining superhigh mechanical frequency, ultrasmall modal mass, and strong optomechanical coupling in a single device.

**Figure 8:**
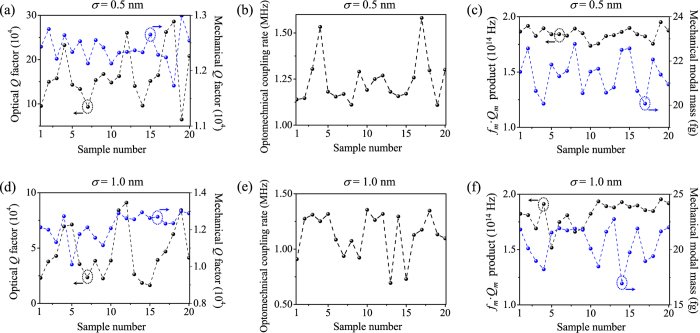
Simulated performance of 20 samples of GaN nanobeam cavity with fabrication imperfections. (**a**–**c**) Effects of randomly varied radii and positions of all air holes (*σ* = 0.5 nm) on the optical and mechanical *Q* factors (**a**), optomechanical coupling rate (**b**), *f*_*m*_·*Q*_*m*_ product and mechanical modal mass (**c**). (**d**–**f**) Effects of randomly varied radii and positions of all air holes (*σ* = 1.0 nm) on the optical and mechanical *Q* factors (**d**), optomechanical coupling rate (**e**), *f*_*m*_·*Q*_*m*_ product and mechanical modal mass (**f**).

To fabricate such a GaN OMC nanobeam cavity, one may adopt the fabrication method in ref. 59. The designed pattern is first transferred to a SiO_2_ hardmask with high-resolution electron-beam lithography with a resist ZEP520A. The SiO_2_ hardmask is then used during the etching of GaN using chlorine-based inductively coupled plasma. Finally the SiO_2_ hardmask is removed by a hydrofluoric acid solution and the GaN nanobeam is released from the substrate by selective etching. Next we study the tolerance to fabrication imperfections of our proposed GaN nanobeam cavities. The statistical evaluation on fabrication disorder^60^,^61^ has concluded that the standard deviation of the air holes’ positions and radii in the fabricated photonic crystal nanocavities is less than 0.58 nm. Therefore, we impose random variations satisfying a normal distribution with a standard deviation *σ* on both radii and center positions of all the air holes of the optimized cavity. Figure 8 lists the simulated optical *Q* factor, mechanical *Q* factor, optomechanical coupling rate, *f*_*m*_·*Q*_*m*_ product, and effective mass for 20 samples with *σ* = 0.5 nm and 1.0 nm, respectively. The average values of the simulated *Q*_*o*_, *Q*_*m*_, *g*_0_/2π, *f*_*m*_·*Q*_*m*_, and *m*_eff_ are 1.64 × 10^5^, 1.24 × 10^4^, 1.24 MHz, 1.85 × 10^14^ Hz, and 21.38 fg when *σ* is 0.5 nm, and are 4.57 × 10^4^, 1.22 × 10^4^, 1.13 MHz, 1.82 × 10^14^ Hz, and 20.57 fg when *σ* is 1.0 nm. A comparison with those of the optimized cavity concludes that *Q*_*m*_, *g*_0_/2π, and *f*_*m*_·*Q*_*m*_ decrease slightly while *Q*_*o*_ is more sensitive to fabrication errors due to the increased reflection loss from the OMC mirrors. A recent paper^62^ reports an experimentally demonstrated *Q_o_* of 55,000 at the wavelength of 788.35 nm in GaN ring resonators with the absorption coefficient (*α*) of ~60 cm^−1^ (ref. 63). The absorption coefficient becomes ~220 cm^−1^ at our designed wavelength of 393.03 nm (ref. 63). According to the relation 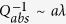 (ref. 59), the *Q*_*abs*_ at 393.03 nm is expected to decrease by 1.82 times from that at 788.35 nm. Due to GaN being an optically active material, its spontaneous emission spectrum can cover the wavelength range of 355–410 nm (ref. 27). The broadband tunable optical gain can also be obtained by incorporating In_*x*_Ga_1−*x*_N fragmented quantum wells^64^. Therefore, the slight material-limited absorption loss could be eliminated by optically pumping the material to its transparency level^65^.

In conclusion, we investigated for the first time ultraviolet OMC cavities based on GaN, for obtaining high optomechanical coupling rate during device miniaturization. With a novel design and optimization strategy based on tuning the OMC mirrors, we can improve the modal confinement as well as the spatial overlap between the optical and mechanical modes, which enables the simultaneous achievement of high *Q* factors, high mechanical frequency, ultrasmall modal mass, and high optomechanical coupling rate. For the optimized OMC nanobeam cavity, the modal mass 22.83 fg is comparable with the designed values of the world’s smallest optomechanical systems^51^,^52^, yet a high optomechanical coupling rate greater than 1.00 MHz is also achieved. The *f*_*m*_·*Q*_*m*_ product is in the 10^14^ Hz regime. Our design and optimization procedure specifies the motivation and objectives clearly in each step, and avoids the blind comprehensive search in the entire space of structural parameters. Therefore, our approach can serve as the general guidelines for developing high-performance OMC nanobeam cavities at other wavelengths or in other materials.

## Methods

The photonic band diagrams are computed using MPB, where a supercell with a size of *a*_*i*_ × 1.00 × 1.00 μm^3^ is encapsulated with periodic boundary conditions. The phononic band diagrams are computed using the structural mechanics module in COMSOL, with the two faces normal to the *x* direction imposed with the Floquet periodic boundary conditions and all the other faces free to move. The reflection spectrum of the OMC mirror is calculated with Lumerical FDTD Solutions^66^ by incorporating the material dispersion of GaN. The radiation-limited optical *Q* factors are computed using MEEP. The computation domain has a total size of 6.00 × 1.40 × 1.20 μm^3^, which includes the entire nanobeam cavity with the surrounding air and perfectly matched layers with a thickness of 0.40 μm. The clamping-loss-limited mechanical *Q* factors are obtained in COMSOL by computing the ratio of the real part and twice of the imaginary part of the complex modal frequency of the mechanical mode following a method described in ref. 67. The following photoelastic constants are employed during calculation of the optomechanical coupling rates^50^: *p*_11_ = *p*_22_ = 0.031, *p*_33_ = 0.033, *p*_12_ = *p*_21_ = 0.008, *p*_13_ = *p*_31_ = 0.006, *p*_23_ = *p*_32_ = 0.006, *p*_44_ = 0.010, *p*_55_ = *p*_66_ = 0.012. The OMC cavity is simulated in COMSOL simultaneously with the electromagnetic waves and structural mechanics modules under the same mesh to obtain the surface and volume integrals in Eqs (2) and (3).

## Additional Information

**How to cite this article**: Zhou, W. *et al.* Ultraviolet optomechanical crystal cavities with ultrasmall modal mass and high optomechanical coupling rate. *Sci. Rep.* **6**, 37134; doi: 10.1038/srep37134 (2016).

**Publisher's note:** Springer Nature remains neutral with regard to jurisdictional claims in published maps and institutional affiliations.

## Figures and Tables

**Table 1:** Optomechanical coupling rate from the five individual contributions *g*_MB_, *g*_PE1_, *g*_PE2_, *g*_PE3_, and *g*_PE4_, for different scaling factors.

Scaling factor	*g*_MB_/2π (kHz)	*g*_PE1_/2π (kHz)	*g*_PE2_/2π (kHz)	*g*_PE3_/2π (kHz)	*g*_PE4_/2π (kHz)
0.90	−248.77	76.11	62.53	1775.25	−0.08
0.95	−334.35	67.32	67.32	1458.30	−0.06
1.00	−357.26	56.21	52.70	1139.28	−0.04

**Table 2:** Comparison on simulated performance of the state-of-the-art OMC nanobeam cavities in three different materials with different optimization methods.

Reference	8	18	This work
Material	Si	Diamond	GaN
Δ*E* (eV)	1.12	5.50	3.40
Young’s modulus (GPa)	(*C*_11_, *C*_12_, *C*_14_) = (166, 64, 80)	1035	330
*λ*_res_ (nm)	1546.40	~740.00	393.03
*f*_*m*_ (GHz)	5.10	12.40	14.97
*m*_eff_ (fg)	136.00	143.00	22.83
*Q*_*o*_	2.20 × 10^7^	2.40 × 10^7^	2.26 × 10^7^
*Q*_*m*_	6.80 × 10^5^ (experimental, at 10 K)	9.10 × 10^6^ ( 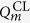 )	1.18 × 10^7^ ( 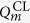 ) 3.78 × 10^5^ (at 10 K)
*g*_0_/2π (MHz)	0.86	1.50	1.26
*V*_*o*_ (*λ*_res_/*n*)^3^	not provided	2.00	0.54
*V*_*m*_ (*λ*_sound_)^3^	0.010	0.045	0.025
Optimization method	Nelder–Mead algorithm	Varying the number of air holes	Tuning the OMC mirrors
